# Supplementary material for: Dynamic transcriptomic profiles of zebrafish gills in response to zinc supplementation
Source: BMC Genomics. 2010 Oct 11;11:553. doi: 10.1186/1471-2164-11-553 (PMC3091702; doi:10.1186/1471-2164-11-553)
Supplement: Additional file 2 — Interactive Direct Interaction Network representing the molecular interactions between zinc, copper, iron, calcium and proteins encoded by transcripts changed by zinc supplementation. Mini web-site containing index.html and hyperlinked pages in subdirectory describing a Direct Interaction Network automatically generated based on curated interactions contained within the proprietary PathwayArchitect database. Ovals represent proteins and the circles symbolize metal ions. Objects are coloured by their abundance in zebrafish at the time-point they were significantly different from the control is a scale from -4 fold (dark green) to +4 fold (dark red). Where significant differences were found at more than one time-point, the colour overlay shows expression at the first instance. Dark blue squares denote 'binding', and light blue squares 'expression'; green squares stand for 'regulation', green diamonds for 'metabolism', and green circles for 'promoter binding'. Arrow heads indicate directionality of the interaction where annotated. All nodes and edges can be further interrogated by selecting the relative area of the image. [file 1471-2164-11-553-S2.zip › PathwayArchitect Zn xs DIN/153038.html]

# PROTEIN: CHEK2

|  |  |
| --- | --- |
| Name | CHEK2 |
| Type | PROTEIN |
| Description | CHK2 checkpoint homolog (S. pombe) |
| Note | In response to DNA damage and replication blocks, cell cycle progression is halted through the control of critical cell cycle regulators. The protein encoded by this gene is a cell cycle checkpoint regulator and putative tumor suppressor. It contains a forkhead-associated protein interaction domain essential for activation in response to DNA damage and is rapidly phosphorylated in response to replication blocks and DNA damage. When activated, the encoded protein is known to inhibit CDC25C phosphatase, preventing entry into mitosis, and has been shown to stabilize the tumor suppressor protein p53, leading to cell cycle arrest in G1. In addition, this protein interacts with and phosphorylates BRCA1, allowing BRCA1 to restore survival after DNA damage. Mutations in this gene have been linked with Li-Fraumeni syndrome, a highly penetrant familial cancer phenotype usually associated with inherited mutations in TP53. Also, mutations in this gene are thought to confer a predisposition to sarcomas, breast cancer, and brain tumors. This nuclear protein is a member of the CDS1 subfamily of serine/threonine protein kinases. Three transcript variants encoding different isoforms have been found for this gene. |
| Alias | HuCds1 |
|  | CHK2 |
|  | Cds1 homolog (S. pombe) |
|  | Rad53 |
|  | serine/threonine-protein kinase CHK2 |
|  | Chek2 |
|  | HUCDS1 |
|  | PP1425 |
|  | Cds1 |
|  | protein kinase CHK2 |
|  | Rad53 homolog (S. cerevisiae) |
|  | CHK2 (checkpoint, S.pombe) homolog |
|  | RAD53 |
|  | checkpoint-like protein CHK2 |
|  | protein kinase Chk2 |
|  | CDS1 |
|  | OTTHUMP00000028871 |
|  | Chk2 |
|  | CHK2 checkpoint homolog |


---

|  |  |
| --- | --- |
| GO Component | nucleus |
|  | membrane |


---

|  |  |
| --- | --- |
| GO ID | GO:0000287 |
|  | GO:0016020 |
|  | GO:0005634 |
|  | GO:0005524 |
|  | GO:0016301 |
|  | GO:0000077 |
|  | GO:0006468 |
|  | GO:0004713 |
|  | GO:0046872 |
|  | GO:0004674 |
|  | GO:0000166 |
|  | GO:0004672 |
|  | GO:0016740 |
|  | GO:0006974 |
|  | GO:0007049 |


---

|  |  |
| --- | --- |
| MIM | MIM:114480 |
|  | MIM:259500 |
|  | MIM:176807 |
|  | MIM:151623 |
|  | MIM:604373 |


---

|  |  |
| --- | --- |
| Connectivity | 231 |


---

|  |  |
| --- | --- |
| Entrez ID | 114212 |
|  | 11200 |
|  | 50883 |


---

|  |  |
| --- | --- |
| Agilent ID | A\_53\_P119711 |
|  | A\_52\_P117090 |
|  | A\_51\_P137111 |
|  | A\_23\_P109453 |
|  | A\_44\_P1032161 |
|  | A\_23\_P109452 |
|  | A\_14\_P137055 |
|  | A\_42\_P605265 |
|  | A\_14\_P104718 |
|  | A\_53\_P115634 |


---

|  |  |
| --- | --- |
| Cellular Localization | Membrane |
|  | Nucleus |
|  | Cell |
|  | Organelle |


---

|  |  |
| --- | --- |
| DbXref | KEGG pathway##04110##Cell cycle##http://www.genome.jp/dbget-bin/show\_pathway?hsa04110+11200 |
|  | Reactome##69620##Cell Cycle Checkpoints##http://www.reactome.org/cgi-bin/eventbrowser?DB=gk\_current&ID=69620 |
|  | KEGG pathway##04110##Cell cycle##http://www.genome.jp/dbget-bin/show\_pathway?rno04110+114212 |
|  | KEGG pathway##04110##Cell cycle##http://www.genome.jp/dbget-bin/show\_pathway?mmu04110+50883 |


---

|  |  |
| --- | --- |
| Pathway | Zn xs inventory |
|  | Zn xs DIN |


---

|  |  |
| --- | --- |
| GO Process | protein amino acid phosphorylation |
|  | cell cycle |
|  | DNA damage checkpoint |
|  | response to DNA damage stimulus |


---

|  |  |
| --- | --- |
| UniGene | Hs.291363 |
|  | Hs.182898 |
|  | Rn.18487 |
|  | Mm.279308 |


---

|  |  |
| --- | --- |
| Affymetrix Probeset ID | 1369010\_at |
|  | 1422747\_at |
|  | 210416\_s\_at |
|  | 37887\_at |
|  | 79039\_at |
|  | 92481\_at |
|  | g13278893\_3p\_a\_at |
|  | rc\_AI071512\_at |
|  | 67276\_at |
|  | TC30457\_at |


---

|  |  |
| --- | --- |
| EC Number | EC 2.7.1.37 |


---

|  |  |
| --- | --- |
| GO Function | protein-tyrosine kinase activity |
|  | protein serine/threonine kinase activity |
|  | magnesium ion binding |
|  | transferase activity |
|  | protein kinase activity |
|  | nucleotide binding |
|  | ATP binding |
|  | kinase activity |
|  | metal ion binding |


---

|  |  |
| --- | --- |
| Nucleotide | AY551305 |
|  | AY551300 |
|  | AY551295 |
|  | AK169781 |
|  | BC004207 |
|  | NM\_016681 |
|  | CR456418 |
|  | NM\_007194 |
|  | AF086904 |
|  | AY551299 |
|  | AF086905 |
|  | NM\_053677 |
|  | AY800241 |
|  | AY551303 |
|  | AK170235 |
|  | BE856024 |
|  | AF174135 |
|  | AY551296 |
|  | AF134054 |
|  | AF217975 |
|  | BC056617 |
|  | AY551302 |
|  | AJ783839 |
|  | AY551298 |
|  | AL121825 |
|  | AJ131197 |
|  | NM\_001005735 |
|  | AY551301 |
|  | AL117330 |
|  | AF096279 |
|  | NM\_145862 |
|  | AB040105 |
|  | AK044913 |
|  | AY551297 |
|  | AY551304 |


---

|  |  |
| --- | --- |
| Protein | AAH56617 |
|  | NP\_446129 |
|  | CAH73823 |
|  | CAH04270 |
|  | AAG17218 |
|  | NP\_009125 |
|  | AAD55890 |
|  | AAS58461 |
|  | AAS58462 |
|  | AAS58458 |
|  | CAA10319 |
|  | AAC83694 |
|  | AAS58459 |
|  | AAS58457 |
|  | O96017 |
|  | NP\_665861 |
|  | Q9Z265 |
|  | BAC32138 |
|  | NP\_001005735 |
|  | AAC83693 |
|  | CAG30304 |
|  | AAV41895 |
|  | AAS58465 |
|  | BAB17231 |
|  | AAS58464 |
|  | AAD11784 |
|  | AAS58463 |
|  | AAS58456 |
|  | NP\_057890 |
|  | CAH73875 |
|  | AAS58466 |
|  | AAS58460 |
|  | AAH04207 |
|  | AAD48504 |


---

|  |  |
| --- | --- |
| Organism | Mammal |


---

|  |  |
| --- | --- |
| Location | chromosome 22, 22q11, 22q11 (Homo sapiens) |
|  | chromosome 12, 12q16 (Rattus norvegicus) |
|  | 22q12.1 (Homo sapiens) |
|  | chromosome 5, 5 F (Mus musculus) |


---

|  |  |
| --- | --- |
